# Genome sequence of the clover-nodulating *Rhizobium leguminosarum* bv. *trifolii* strain SRDI565.

**DOI:** 10.4056/sigs.4468250

**Published:** 2013-12-15

**Authors:** Wayne Reeve, Elizabeth Drew, Ross Ballard, Vanessa Melino, Rui Tian, Sofie De Meyer, Lambert Brau, Mohamed Ninawi, Hazuki Teshima, Lynne Goodwin, Patrick Chain, Konstantinos Liolios, Amrita Pati, Konstantinos Mavromatis, Natalia Ivanova, Victor Markowitz, Tanja Woyke, Nikos Kyrpides

**Affiliations:** 1 Centre for Rhizobium Studies, Murdoch University, Western Australia, Australia; 2 South Australian Research and Development Institute, Urrbrae, South Australia, Australia; 3 School of Life and Environmental Sciences, Faculty of Science & Technology, Deakin University, Melbourne, Victoria, Australia; 4 Los Alamos National Laboratory, Bioscience Division, Los Alamos, New Mexico, USA; 5 DOE Joint Genome Institute, Walnut Creek, California, USA; 6 Biological Data Management and Technology Center, Lawrence Berkeley National Laboratory, Berkeley, California, USA

**Keywords:** root-nodule bacteria, nitrogen fixation, rhizobia, *Alphaproteobacteria*

## Abstract

*Rhizobium leguminosarum* bv. *trifolii* SRDI565 (syn. N8-J) is an aerobic, motile, Gram-negative, non-spore-forming rod. SRDI565 was isolated from a nodule recovered from the roots of the annual clover *Trifolium subterraneum* subsp. *subterraneum* grown in the greenhouse and inoculated with soil collected from New South Wales, Australia. SRDI565 has a broad host range for nodulation within the clover genus, however N_2_-fixation is sub-optimal with some *Trifolium* species and ineffective with others. Here we describe the features of *R. leguminosarum* bv. *trifolii* strain SRDI565, together with genome sequence information and annotation. The 6,905,599 bp high-quality-draft genome is arranged into 7 scaffolds of 7 contigs, contains 6,750 protein-coding genes and 86 RNA-only encoding genes, and is one of 100 rhizobial genomes sequenced as part of the DOE Joint Genome Institute 2010 Genomic Encyclopedia for Bacteria and Archaea-Root Nodule Bacteria (GEBA-RNB) project.

## Introduction

Plant available nitrogen is a precious commodity in many agricultural soils and the most commonly limiting nutrient in plant growth. The supply of plant available nitrogen to nitrogen (N)-deficient farming systems is thus vital to productivity [1]. The application of industrially fixed nitrogenous fertilizer can meet the demand for N. However, this is a costly option as the price of nitrogenous fertilizer is connected to the cost of fossil fuels required for its production. Furthermore, the use of nitrogenous fertilizer contributes to greenhouse gas emissions and pollution of the environment. A more environmentally sustainable option is to exploit the process of biological nitrogen fixation that occurs in the symbiosis between legumes and rhizobia [2].

In this symbiotic association, rhizobia reduce atmospheric dinitrogen (N_2_) into bioavailable N that can be used by the plant for growth. Pasture legumes, including the clovers that comprise the *Trifolium* genus, are major contributors of biologically fixed N_2_ to mixed farming systems throughout the world [3,4]. In Australia, soils with a history of growing *Trifolium* spp. have developed large and symbiotically diverse populations of *Rhizobium leguminosarum* bv. *trifolii* (*R. l. trifolii*) that are able to infect and form nodules on a range of clover species. The N_2_-fixation capacity of the symbioses established by different combinations of clover hosts (*Trifolium* spp.) and strains of *R. l. trifolii* can vary from 10 to 130% when compared to an effective host-strain combination [3-9].

*R. l. trifolii* strain SRDI565 (syn. N8-J [10]) was isolated from a nodule recovered from the roots of the annual clover *Trifolium subterraneum* subsp. *subterraneum* that had been inoculated with soil collected from under a mixed pasture stand from Tumet, New South Wales, Australia and grown in N deficient media for four weeks after inoculation, in the greenhouse. SRDI565 was first noted for its sub-optimal N_2_-fixation capacity on *T. subterraneum* cv. Campeda (<60% of that with strain WSM1325) and formation of white (Fix-) pseudo-nodules on *T. subterraneum* cv. Clare [10,11]. Here we present a preliminary description of the general features for *R. leguminosarum* bv. *trifolii* strain SRDI565 together with its genome sequence and annotation.

## Classification and general features

**Figure 1:**
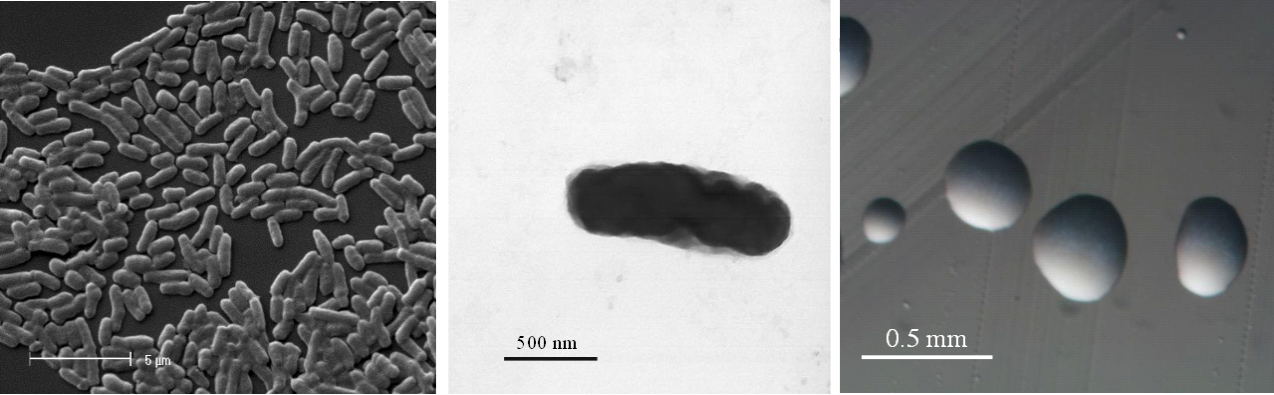
Images of *Rhizobium leguminosarum* bv. *trifolii* strain SRDI565 using scanning (Left) and transmission (Center) electron microscopy as well as light microscopy to show the colony morphology on solid media (Right).

*R. l. trifolii* strain SRDI565 is a motile, Gram-negative rod (Figure 1 Left and Center) in the order *Rhizobiales* of the class *Alphaproteobacteria*. It is fast growing, forming colonies within 3-4 days when grown on half strength Lupin Agar (½LA) [12] at 28°C. Colonies on ½LA are white-opaque, slightly domed and moderately mucoid with smooth margins (Figure 1 Right).

**Figure 2:**
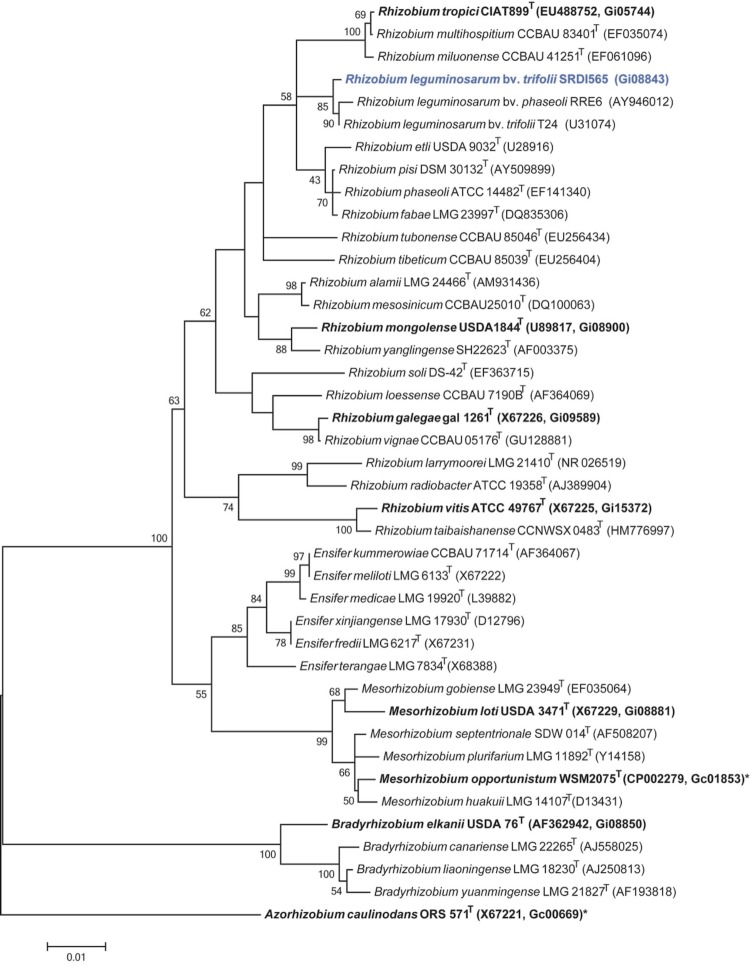
Phylogenetic tree showing the relationship of *Rhizobium leguminosarum* bv. *trifolii* SRDI565 (shown in blue print) with some of the root nodule bacteria in the order *Rhizobiales* based on aligned sequences of the 16S rRNA gene (1,307 bp internal region). All sites were informative and there were no gap-containing sites. Phylogenetic analyses were performed using MEGA, version 5.05 [28]. The tree was built using the maximum likelihood method with the General Time Reversible model. Bootstrap analysis [29] with 500 replicates was performed to assess the support of the clusters. Type strains are indicated with a superscript T. Strains with a genome sequencing project registered in GOLD [30] are in bold print and the GOLD ID is shown after the accession number. Published genomes are indicated with an asterisk.

Minimum Information about the Genome Sequence (MIGS) is provided in Table 1. Figure 2 shows the phylogenetic neighborhood of *R. l. trifolii* strain SRDI565 in a 16S rRNA sequence based tree. This strain clusters closest to *R. l. trifolii* T24 and *Rhizobium leguminosarum* bv. *phaseoli* RRE6 with 99.8% and 99.6% sequence identity, respectively.

**Table 1:** Classification and general features of *Rhizobium leguminosarum* bv. *trifolii* SRDI565 according to the MIGS recommendations [13]

**MIGS ID**	**Property**	**Term**	**Evidence code**
	Current classification	Domain *Bacteria*	TAS [13,14]
		Phylum *Proteobacteria*	TAS [15]
		Class *Alphaproteobacteria*	TAS [16]
		Order *Rhizobiales*	TAS [17,18]
		Family *Rhizobiaceae*	TAS [19,20]
		Genus *Rhizobium*	TAS [19,21-24]
		Species *Rhizobium leguminosarum* bv. *trifolii*	TAS [19,21,24,25]
	
	Gram stain	Negative	IDA
	Cell shape	Rod	IDA
	Motility	Motile	IDA
	Sporulation	Non-sporulating	NAS
	Temperature range	Mesophile	NAS
	Optimum temperature	28°C	NAS
	Salinity	Non-halophile	NAS
MIGS-22	Oxygen requirement	Aerobic	TAS [11]
	Carbon source	Varied	NAS
	Energy source	Chemoorganotroph	NAS
MIGS-6	Habitat	Soil, root nodule, on host	TAS [10]
MIGS-15	Biotic relationship	Free living, symbiotic	TAS [10]
MIGS-14	Pathogenicity	Non-pathogenic	NAS
	Biosafety level	1	TAS [26]
	Isolation	Root nodule	TAS [10]
MIGS-4	Geographic location	NSW, Australia	TAS [10]
MIGS-5	Soil collection date	Dec, 1998	IDA
MIGS-4.1 MIGS-4.2	Longitude Latitude	148.25 -35.32	IDA
MIGS-4.3	Depth	0-10cm	
MIGS-4.4	Altitude	Not recorded	

Evidence codes – IDA: Inferred from Direct Assay; TAS: Traceable Author Statement (i.e., a direct report exists in the literature); NAS: Non-traceable Author Statement (i.e., not directly observed for the living, isolated sample, but based on a generally accepted property for the species, or anecdotal evidence). These evidence codes are from the Gene Ontology project [27].

### Symbiotaxonomy

*R. l. trifolii* SRDI565 forms nodules on (Nod^+^), and fixes N_2_ (Fix^+^) with, a range of annual and perennial clover species of Mediterranean origin (Table 2). SRDI565 forms white, ineffective (Fix^-^) nodules with annual clovers *T. glanduliferum* and *T. subterraneum* cv. Clare, and with the perennial clovers *T. pratense and T. polymorphum*. SRDI565 does not form nodules on *T. vesiculosum*.

**Table 2:** Compatibility of SRDI565 with eleven *Trifolium* genotypes for nodulation (Nod) and N_2_-Fixation (Fix)

**Species name**	**Cultivar**	**Common Name**	**Growth Type**	**Nod**	**Fix**	**Reference**
*T. glanduliferum* Boiss.	Prima	Gland	Annual	+(w)	-	
*T. michelianum* Savi.	Bolta	Balansa	Annual	+	+	
*T. purpureum* Loisel	Paratta	Purple	Annual	+	+	[11]
*T. resupinatum* L.	Kyambro	Persian	Annual	+	+	
*T. subterraneum* L.	Campeda	Sub. clover	Annual	+	+	[10,11]
*T. subterraneum* L.	Clare	Sub. clover	Annual	+(w)	-	[10,11]
*T. vesiculosum* Savi.	Arrotas	Arrowleaf	Annual	-	-	
*T. fragiferum* L.	Palestine	Strawberry	Perennial	+	+	
*T. polymorphum* Poir	Acc.#087102	Polymorphous	Perennial	+(w)	-	[11]
*T. pratense* L.	-	Red	Perennial	+(w)	-	
*T. repens* L.	Haifa	White	Perennial	+	+	

(w) indicates nodules present were white.

## Genome sequencing and annotation information

### Genome project history

This organism was selected for sequencing on the basis of its environmental and agricultural relevance to issues in global carbon cycling, alternative energy production, and biogeochemical importance, and is part of the Community Sequencing Program at the U.S. Department of Energy, Joint Genome Institute (JGI) for projects of relevance to agency missions. The genome project is deposited in the Genomes OnLine Database [30] and an improved-high-quality-draft genome sequence in IMG. Sequencing, finishing and annotation were performed by the JGI. A summary of the project information is shown in Table 3.

**Table 3:** Genome sequencing project information for *Rhizobium leguminosarum* bv. *trifolii* strain SRDI565.

**MIGS ID**	**Property**	**Term**
MIGS-31	Finishing quality	Improved high-quality draft
MIGS-28	Libraries used	2× Illumina libraries; Std short PE & CLIP long PE
MIGS-29	Sequencing platforms	Illumina HiSeq 2000, PacBio
MIGS-31.2	Sequencing coverage	862× Illumina
MIGS-30	Assemblers	with Allpaths, version 39750, Velvet 1.015, phrap 4.24
MIGS-32	Gene calling methods	Prodigal 1.4, GenePRIMP
	GOLD ID	Gi08843
	NCBI project ID	81743
	Database: IMG	2517287029
	Project relevance	Symbiotic N_2_ fixation, agriculture

### Growth conditions and DNA isolation

*Rhizobium leguminosarum* bv. *trifolii* strain SRDI565 was cultured to mid logarithmic phase in 60 ml of TY rich media [31] on a gyratory shaker at 28°C. DNA was isolated from the cells using a CTAB (Cetyl trimethyl ammonium bromide) bacterial genomic DNA isolation method [32].

### Genome sequencing and assembly

The genome of *Rhizobium leguminosarum* bv. *trifolii* strain SRDI565 was sequenced at the Joint Genome Institute (JGI) using Illumina [33] data. An Illumina short-insert paired-end library with an average insert size of 243 + 58 bp was used to generate 18,700,764 reads and an Illumina long-insert paired-end library with an average insert size of 8,446 + 2,550 bp was used to generate 21,538,802 reads totalling 6,036 Mbp of Illumina data (unpublished, Feng Chen).

All general aspects of library construction and sequencing performed at the JGI can be found at the JGI user homepage [34]. The initial draft assembly contained 22 contigs in 16 scaffolds. The initial draft data was assembled with Allpaths, version 39750, and the consensus was computationally shredded into 10 Kb overlapping fake reads (shreds). The Illumina draft data was also assembled with Velvet, version 1.1.05 [35], and the consensus sequences were computationally shredded into 1.5 Kb overlapping fake reads (shreds). The Illumina draft data was assembled again with Velvet using the shreds from the first Velvet assembly to guide the next assembly. The consensus from the second VELVET assembly was shredded into 1.5 Kb overlapping fake reads. The fake reads from the Allpaths assembly and both Velvet assemblies and a subset of the Illumina CLIP paired-end reads were assembled using parallel phrap, version 4.24 (High Performance Software, LLC). Possible mis-assemblies were corrected with manual editing in Consed [36-38]. Gap closure was accomplished using repeat resolution software (Wei Gu, unpublished), and sequencing of bridging PCR fragments with PacBio (unpublished, Cliff Han) technology. For improved high quality draft, 4 PCR PacBio consensus sequences were completed to close gaps and to raise the quality of the final sequence. The estimated total size of the genome is 7 Mb and the final assembly is based on 6,036 Mb of Illumina draft data, which provides an average 862× coverage of the genome.

### Genome annotation

Genes were identified using Prodigal [39] as part of the DOE-JGI annotation pipeline [40], followed by a round of manual curation using the JGI GenePRIMP pipeline [41]. The predicted CDSs were translated and used to search the National Center for Biotechnology Information (NCBI) non-redundant database, UniProt, TIGRFam, Pfam, PRIAM, KEGG, COG, and InterPro databases. These data sources were combined to assert a product description for each predicted protein. Non-coding genes and miscellaneous features were predicted using tRNAscan-SE [42], RNAMMer [43], Rfam [44], TMHMM [45], and SignalP [46]. Additional gene prediction analyses and functional annotation were performed within the Integrated Microbial Genomes (IMG-ER) platform [47,48].

## Genome properties

**Figure 3:**
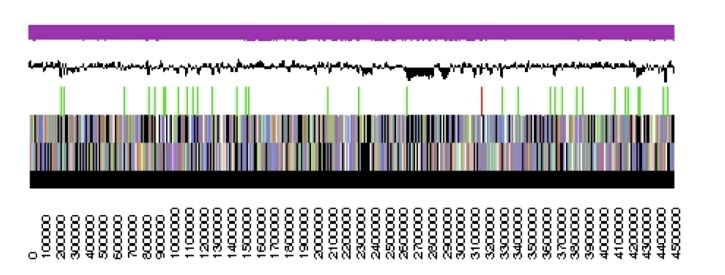
Graphical map of the genome of *Rhizobium leguminosarum* bv. *trifolii* strain SRDI565 (scaffold 1.1). From bottom to the top of each scaffold: Genes on forward strand (color by COG categories as denoted by the IMG platform), Genes on reverse strand (color by COG categories), RNA genes (tRNAs green, sRNAs red, other RNAs black), GC content, GC skew.

**Figure 4:**
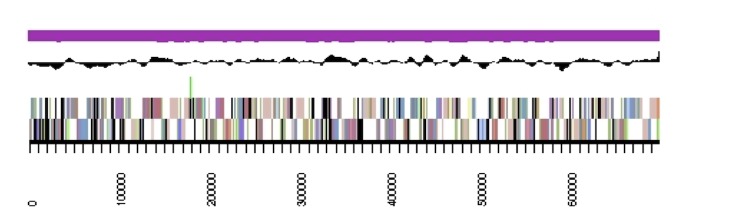
Graphical map of the genome of *Rhizobium leguminosarum* bv. *trifolii* strain SRDI565 (scaffold 2.2). From bottom to the top of each scaffold: Genes on forward strand (color by COG categories as denoted by the IMG platform), Genes on reverse strand (color by COG categories), RNA genes (tRNAs green, sRNAs red, other RNAs black), GC content, GC skew.

**Figure 5:**
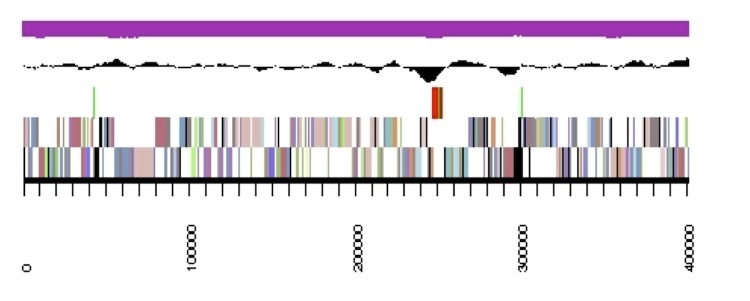
Graphical map of the genome of *Rhizobium leguminosarum* bv. *trifolii* strain SRDI565 (scaffold 3.3). From bottom to the top of each scaffold: Genes on forward strand (color by COG categories as denoted by the IMG platform), Genes on reverse strand (color by COG categories), RNA genes (tRNAs green, sRNAs red, other RNAs black), GC content, GC skew.

**Figure 6:**
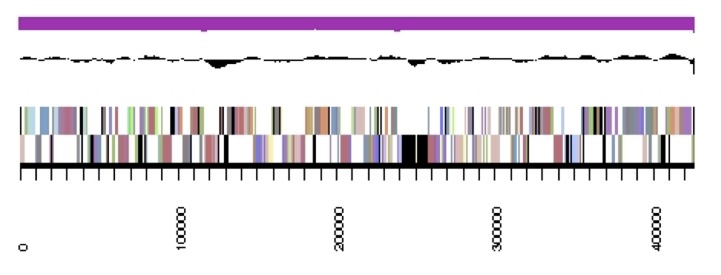
Graphical map of the genome of *Rhizobium leguminosarum* bv. *trifolii* strain SRDI565 (scaffold 4.4). From bottom to the top of each scaffold: Genes on forward strand (color by COG categories as denoted by the IMG platform), Genes on reverse strand (color by COG categories), RNA genes (tRNAs green, sRNAs red, other RNAs black), GC content, GC skew.

**Figure 7:**
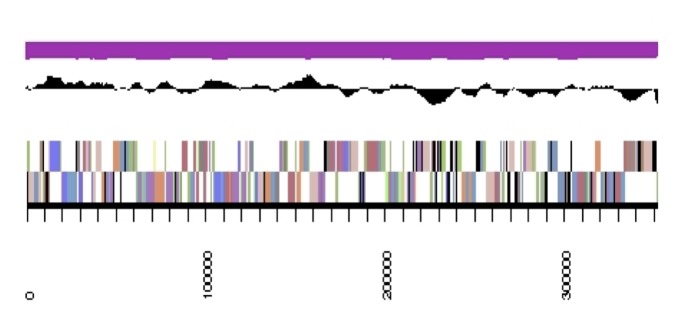
Graphical map of the genome of *Rhizobium leguminosarum* bv. *trifolii* strain SRDI565 (scaffold 5.5). From bottom to the top of each scaffold: Genes on forward strand (color by COG categories as denoted by the IMG platform), Genes on reverse strand (color by COG categories), RNA genes (tRNAs green, sRNAs red, other RNAs black), GC content, GC skew.

**Figure 8:**
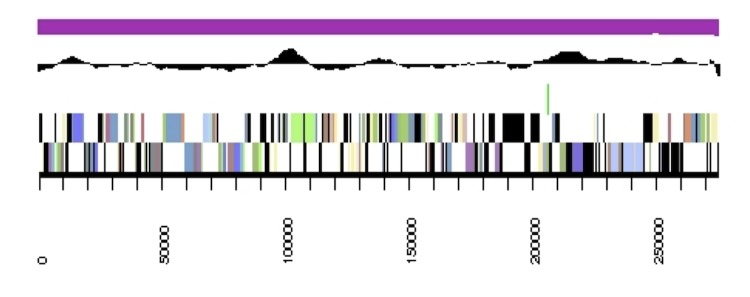
Graphical map of the genome of *Rhizobium leguminosarum* bv. *trifolii* strain SRDI565 (6.6). From bottom to the top of each scaffold: Genes on forward strand (color by COG categories as denoted by the IMG platform), Genes on reverse strand (color by COG categories), RNA genes (tRNAs green, sRNAs red, other RNAs black), GC content, GC skew.

**Figure 9:**
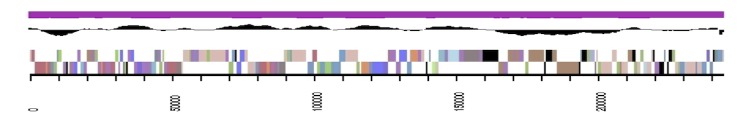
Graphical map of the genome of *Rhizobium leguminosarum* bv. *trifolii* strain SRDI565 (7.7). From bottom to the top of each scaffold: Genes on forward strand (color by COG categories as denoted by the IMG platform), Genes on reverse strand (color by COG categories), RNA genes (tRNAs green, sRNAs red, other RNAs black), GC content, GC skew.

The genome is 6,905,599 nucleotides with 60.67% GC content (Table 4) and comprised of 7 scaffolds (Figures 3,4,5,6,7,8,and 9) of 7 contigs. From a total of 6,836 genes, 6,750 were protein encoding and 86 RNA-only encoding genes. The majority of genes (77.98%) were assigned a putative function whilst the remaining genes were annotated as hypothetical. The distribution of genes into COGs functional categories is presented in Table 5.

**Table 4:** Genome Statistics for *Rhizobium leguminosarum* bv. *trifolii* SRDI565

**Attribute**	**Value**	**% of Total**
Genome size (bp)	6,905,599	100.00
DNA coding region (bp)	5,960,775	86.32
DNA G+C content (bp)	4,189,855	60.67
Number of scaffolds	7	
Number of contigs	7	
Total gene	6,836	100.00
RNA genes	86	1.26
rRNA operons*	3	
Protein-coding genes	6,750	98.74
Genes with function prediction	5,331	77.98
Genes assigned to COGs	5,330	77.97
Genes assigned Pfam domains	5,535	80.97
Genes with signal peptides	603	8.82
Genes with transmembrane helices	1,552	22.70
CRISPR repeats	0	

**Table 5:** Number of protein coding genes of *Rhizobium leguminosarum* bv. *trifolii* SRDI565 associated with the general COG functional categories.

**Code**	**Value**	**%age**	**Description**
J	191	3.22	Translation, ribosomal structure and biogenesis
A	0	0.00	RNA processing and modification
K	574	9.67	Transcription
L	189	3.19	Replication, recombination and repair
B	3	0.05	Chromatin structure and dynamics
D	41	0.69	Cell cycle control, mitosis and meiosis
Y	0	0.00	Nuclear structure
V	70	1.18	Defense mechanisms
T	320	5.39	Signal transduction mechanisms
M	315	5.31	Cell wall/membrane biogenesis
N	81	1.37	Cell motility
Z	0	0.00	Cytoskeleton
W	0	0.00	Extracellular structures
U	96	1.62	Intracellular trafficking and secretion
O	208	3.51	Posttranslational modification, protein turnover, chaperones
C	326	5.49	Energy production conversion
G	633	10.67	Carbohydrate transport and metabolism
E	591	9.96	Amino acid transport metabolism
F	109	1.84	Nucleotide transport and metabolism
H	193	3.25	Coenzyme transport and metabolism
I	216	3.64	Lipid transport and metabolism
P	272	4.58	Inorganic ion transport and metabolism
Q	148	2.49	Secondary metabolite biosynthesis, transport and catabolism
R	758	12.77	General function prediction only
S	600	10.11	Function unknown
-	1,506	22.03	Not in COGS
